# Metabolomics reveals LysoPC a C17:0 (LPC 17:0) as candidate biomarker for personalized medicine in morbid obesity

**DOI:** 10.3389/fmed.2026.1779275

**Published:** 2026-03-30

**Authors:** Eleonora Stefanini, Silvia Marin, Joan Serrano-Marín, Juan Sánchez-Navés, Hanan Awad Alkozi, Mercè Pallàs, Marta Cascante, Christian Griñán-Ferré, Rafael Franco

**Affiliations:** 1 Department of Biochemistry and Molecular Biomedicine, Universitat de Barcelona, Barcelona, Spain; 2 Institute of Biomedicine of University of Barcelona (IBUB), University of Barcelona (UB), Barcelona, Spain; 3 CIBEREHD, Network Center for Hepatic and Digestive Diseases, Spanish National Health Institute Carlos III (ISCIII), Madrid, Spain; 4 Department of Ophthalmology, Oftalmedic, I.P.O. Institute of Ophthalmology, Palma de Mallorca, Spain; 5 Department of Optometry, College of Applied Medical Sciences, Qassim University, Buraydah, Saudi Arabia; 6 Departament de Farmacologia i Química Terapeùtica, Universitat de Barcelona, Barcelona, Spain; 7 Institut de Neurociències, Universitat de Barcelona, Barcelona, Spain; 8 CiberNed, Network Center for Neurodegenerative Diseases, Spanish National Health Institute Carlos III (ISCIII), Madrid, Spain; 9 Institute of Theoretical and Computational Chemistry (IQTC) of the University of Barcelona, Barcelona, Spain

**Keywords:** biomarker, blood, diagnosis, metabolomics, lipidomics, lysophosphatidylcholines, P5 medicine

## Abstract

Morbid obesity represents the most severe form of obesity and is associated with increased cardiometabolic risk, including type 2 diabetes and cardiovascular complications. Current diagnostic approaches rely on anthropometric measures, failing to capture the metabolic heterogeneity among patients. In this study, plasma samples from 20 morbidly obese patients and 8 controls were analyzed using targeted metabolomics. Of 188 quantified metabolites, 139 passed quality control across lipid, amino acid, and acylcarnitine families. Applying a novel normalization strategy, LPC 17:0 emerged as the most consistent discriminative biomarker, achieving 78% accuracy in distinguishing patients from controls. PC O-40:1, selected as the concomitant within the phosphatidylcholine family based on statistical performance, also showed promise, notable for its abundance in heart and liver tissue and its proposed antioxidant role. The difference between predicted and actual LPC 17:0 levels correlated negatively with BMI (r ≈ −0.6), highlighting its value as a marker of obesity severity. While combining LPC 17:0 with other metabolites slightly improved classification, the metabolite alone demonstrated strong discriminative power. These findings introduce a novel biomarker for morbid obesity and support the development of personalized medicine approaches, enabling monitoring of disease progression, improved risk stratification and targeted therapeutic interventions.

## Introduction

Obesity has become one of the most pressing health challenges of the 21st century, with prevalence rates continuing to rise worldwide. The condition is associated with increased risk of type 2 diabetes (T2D), cardiovascular disease, cancer, and neurodegenerative disorders, and represents a major socioeconomic burden (1). Despite decades of research, the molecular mechanisms underlying obesity remain incompletely understood, and strategies for early diagnosis, prevention, and personalized treatment remain limited. The pathophysiology of obesity is marked by dysregulation of multiple interconnected systems, including energy balance, lipid metabolism, inflammation, and endocrine signaling. Morbid obesity, in particular, defined as a body mass index (BMI) > 40 kg/m2 or >35 kg/m2 with comorbidities, is often accompanied by profound metabolic disturbances such as insulin resistance, dyslipidemia, and chronic inflammation, making it a suitable context for the discovery of metabolic biomarkers (2, 3). The multifactorial nature of obesity, shaped by genetic variation, epigenetic changes, environmental exposures, and lifestyle factors, creates substantial heterogeneity that complicates both clinical management and therapeutic development (4, 5).

Metabolomics has emerged as a transformative approach to address this complexity by providing a comprehensive snapshot of biochemical processes within living systems. As the most downstream of the omics technologies, metabolomics captures the functional consequences of upstream genetic, transcriptomic, and proteomic alterations, thereby reflecting the phenotype of disease states (6, 7). Advances in high-resolution mass spectrometry and chromatographic separation now enable the simultaneous quantification of hundreds of metabolites from minimal sample volumes, revolutionizing our capacity to detect subtle biochemical alterations and fueling new directions in biomarker discovery and precision medicine (8).

Within this metabolomic landscape, lysophosphatidylcholines (LPCs) have gained significant attention as key indicators of metabolic health. LPCs act as key carriers of fatty acids and choline in human systemic metabolism (9), and higher circulating levels have been associated with protection against obesity and T2D (10). Their biological influence spans from early development, where cord blood concentrations are linked to neonatal adiposity (11), to adulthood, where low plasma LPCs are associated with impaired mitochondrial oxidative capacity (12). Crucially, a deficit in LPC-mediated *β*-oxidation can lead to the accumulation of toxic ceramides, which impair endothelial nitric oxide production and drive vascular damage (13). Beyond obesity, LPCs are indispensable for maintaining neuronal membrane integrity (14), while their sequestration by tumor cells for membrane biogenesis has been described in various cancers (15).

The integration of metabolomics with machine learning has further advanced its diagnostic potential by enabling the construction of robust multivariate models. Crucially, metabolomics can reveal molecular perturbations that precede clinical onset, offering opportunities for early diagnosis and patient stratification (16, 17). Nonetheless, a major obstacle to the clinical translation of metabolomics lies in the intrinsic variability of human samples, driven by demographic, dietary, genetic, and environmental influences. This biological noise often obscures disease-related signals and limits reproducibility across studies. To address this, we previously developed a novel analytical framework that combines concomitant parameters with residual analysis to minimize inter individual variability (18). Applied to published datasets, this approach enabled the identification of small metabolite combinations capable of distinguishing Alzheimer’s disease patients from controls with accuracies above 75%, thereby demonstrating the value of rigorous normalization and multivariate modeling in biomarker discovery (19).

In the present study, we extend these methodological advances to the context of morbid obesity. Using plasma samples from morbidly obese patients and matched controls, we performed targeted metabolomics coupled with our normalization and feature-selection pipeline. Our objective was to identify metabolite signatures that discriminate obesity-associated diabetes and may support stratified approaches in patient care. Beyond biomarker discovery, the findings aim to contribute to the broader framework of precision medicine in obesity management, where therapeutic decisions for individual patients are guided by their heterogeneous biological and clinical profiles.

## Results

### Correlations between data from patients and controls

#### Cohort overview and data preprocessing

**Figure 1:**
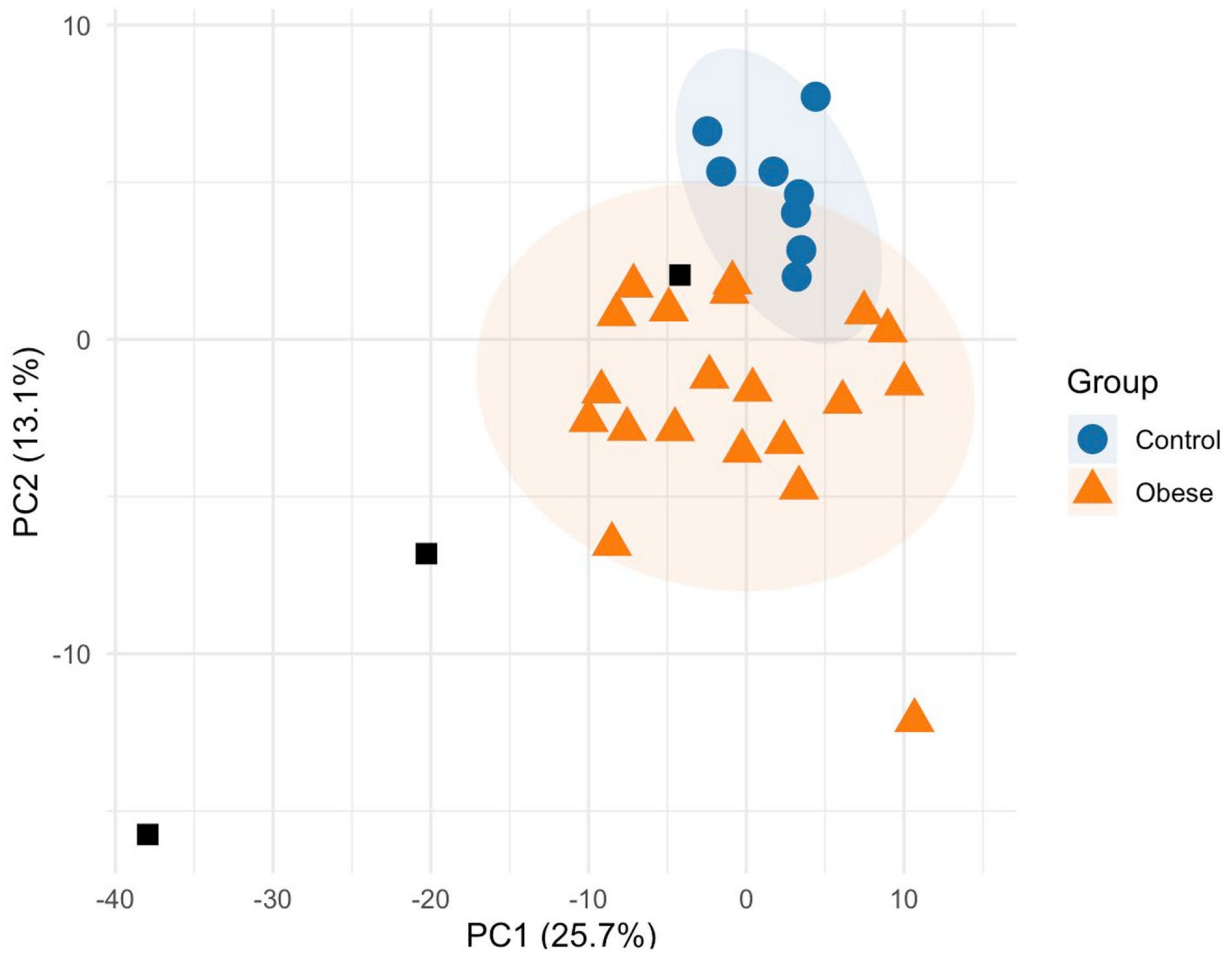
Principal component analysis (PCA) score plot of plasma metabolic profiles. Control subjects are represented by blue circles (*n* = 8) and obese patients by orange triangles (*n* = 20) based on 139 metabolites. Black squares represent the three kit-provided quality control samples (QC1–QC3), shown for reference but not included in the PCA model calculation. As these correspond to different concentration levels of lyophilized controls rather than pooled technical replicates, they are not expected to cluster together in the PCA space. Shaded areas represent 95% confidence ellipses for each group. The plot uses a color-blind friendly palette and redundant shape coding for enhanced accessibility.

The initial dataset included 30 plasma samples (20 -morbid- obese patients and 10 control subjects) containing both lipid and small-molecule metabolite data. Of the 188 initial metabolites, 49 were excluded due to >20% missing values, resulting in a working dataset of 139 metabolites. All 30 samples passed the missingness threshold, and no sample was excluded at this stage. Principal component analysis (PCA) identified two control samples as outliers, leaving a final dataset of 28 samples (20 obese and 8 controls) and 139 metabolites. An exploratory PCA performed on this final dataset (Figure 1) demonstrated a multivariate separation between the groups along the first two principal components, which together accounted for 38.8% (PC1: 25.7%, PC2: 13.1%) of the total variance. PCA was applied as an unsupervised, data-driven exploratory method (with class labels withheld during model fitting), enabling an objective characterization of the global metabolic structure and visualization of emergent between-group separation. As shown in Figure 1, while most subjects clustered within their respective 95% confidence ellipses, the obese group exhibited greater metabolic dispersion, reflecting the inherent clinical heterogeneity of the morbidly obese phenotype. The control group, conversely, exhibited a more homogeneous metabolic profile. Following this exploratory analysis, the 139 metabolites were subsequently grouped into five distinct biochemical categories according to their chemical structure: acylcarnitines (11 metabolites), amino acids (21 metabolites), biogenic amines (7 metabolites), glycerophospholipids (85 metabolites) and sphingomyelins (14 metabolites). One parameter, H1, was excluded as it corresponds to the sum of the level of different molecules (hexoses). This variable was excluded to avoid introducing a non-independent, aggregated signal that could inflate shared variance and obscure metabolite-specific effects, since H1 reflects the summed abundance of multiple hexoses rather than a single molecular feature. A total of 138 metabolites were considered for downstream analysis. Overall, preprocessing and exploratory analyses followed established metabolomics multivariate workflows to maximize interpretability and analytical robustness given the available sample size.

#### Selection of the *metabolite_c* for each biochemical family

Whitin each biochemical family, a concomitant metabolite (*metabolite_c*) was selected according to the procedure described in Methods, yielding a total of 7,894 models. Based on the thresholds of average R^2^ > 0.2, F-test *p*-value < 0.05, and concomitant-specific p-value < 0.05, the following metabolites were chosen as concomitants: decanoylcarnitine (C10) for acylcarnitines, ornithine for amino acids, PC O-40:1 for glycerophospholipids, and SM 16:0 for sphingomyelins (Table 1). The nomenclature suggested by Liebisch et al., has been used along the manuscript (20). Although phenylethylamine emerged as the top hit for biogenic amines, and is therefore reported in Table 1, it did not meet the predefined thresholds; this biochemical group was consequently excluded from further analysis. This selection step was explicitly exploratory and was used solely to nominate candidate adjustment variables for the subsequent models, not to claim independent biological associations.

**Table 1:** Statistical metrics (average) associated to the models with the selected concomitant within each biochemical group.

Biochemical group	Model^a^^,^^b^^,^^c^	Avg *R^2^*	Fisher-*p*-value	*p*-value concomitant
Acylcarnitines	log_10_(metabolite)_predicted_ ~ Group + Age*Sex + Fasting_time + log_10_ [**C10**]	0.35	0.01	0.05
Amino acids	log_10_(metabolite)_predicted_ ~ Group + Age*Sex + Fasting_time + log_10_ [**Ornithine**]	0.27	0.02	0.04
Biogenic amines	log_10_(metabolite)_predicted_ ~ Group + Age*Sex + Fasting_time + log_10_ [**Phenylethylamine**]	0.16	0.05	0.06
Glycerophospholipids	log_10_(metabolite)_predicted_ ~ Group + Age*Sex + Fasting_time + log_10_ [**PC O-40:1**]	0.39	0.0022	0.0056
Sphingomyelins	log_10_(metabolite)_predicted_ ~ Group + Age*Sex + Fasting_time + log_10_ [**SM 18:0**]	0.51	2.09 × 10^−4^	4.47 × 10^−4^

a Group refers to the study group, included as a categorical variable (controls as the reference category).

b Sex is included as a categorical variable (male as the reference category).

c The selected concomitant metabolite (metabolite_c) is in bold.

#### Residual correlation heatmaps

Residual correlation analyses were performed for the four biochemical families with a selected concomitant metabolite (*metabolite_c*), to explore inter-metabolite relationships beyond known covariates. Residuals were computed as the difference between the observed and predicted log_10_-transformed metabolite values (see Methods, Equation 2). Pearson correlation coefficients were calculated on these residuals, and pairwise correlations were visualized in heatmaps. In each plot, metabolite label colors indicate the direction of change in raw data between obese patients and controls (red = tendency to increase in obesity; blue = tendency to decrease in obesity), while the rectangle with solid borders highlight metabolites significantly associated with condition (*p* < 0.05) in the original regression model.

**Figure 2:**
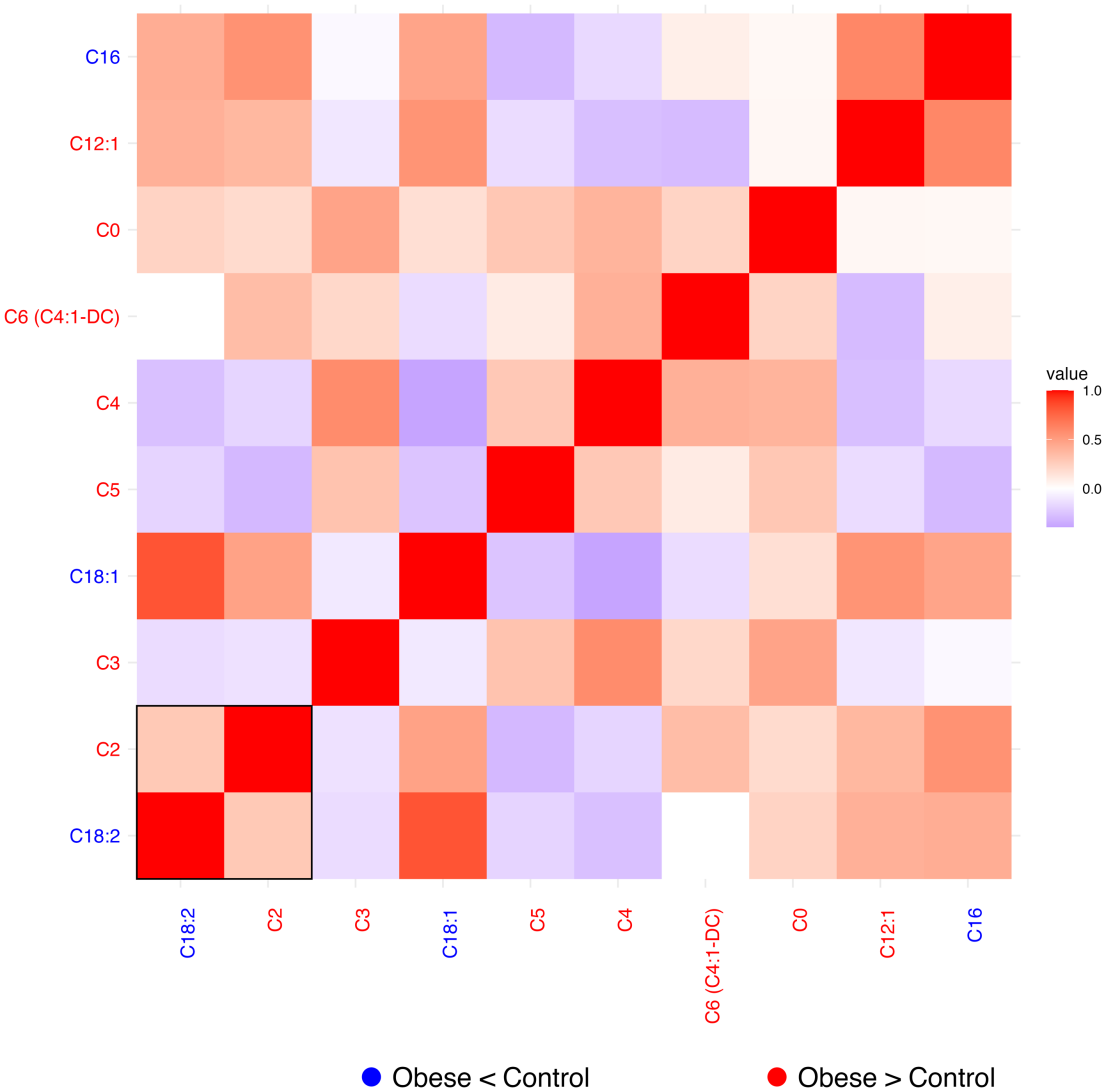
Heatmap of residual correlations within the acylcarnitines group. *Metabolite_c* for this family was C10 acylcarnitine. Blue labels indicate tendency to decrease in obesity and red labels indicate a tendency to increase in obesity. Red cells indicate positive correlation (1 means perfect correlation), blue cells indicate negative correlation, and white cells indicate no correlation. *p* < 0.05 for residuals of metabolites within the solid line frame.

For the acylcarnitine family, the residual correlation heatmap (Figure 2) revealed generally modest pairwise associations, with positive and negative correlations appearing balanced. Most acylcarnitines tended to show higher levels in obese individuals (red labels), consistent with the expected increase in obesity. Notable exceptions included C18:1, C18:2, and C16, which were reduced in obese subjects (blue labels). Residuals of the two metabolites C18:2 and C2, enclosed in a solid box in the table, showed a significant association with condition.

Heatmaps for amino acids, glycerophospholipids, and sphingomyelins are presented in Supplementary Figures S1–S3, respectively. Across these families, some metabolites showed higher levels in samples from obese individuals, while others were reduced. The residual correlations appeared balanced, with both positive and negative correlations. In the amino acid group, significant associations with the condition were limited to glycine (decreased) and glutamine (increased). Several glycerophospholipids showed significant associations with the condition, reflecting the large size of this family. For sphingomyelins, significance was observed for SM 26:1 (decreased) and for SM 16:1 and SM 18:1 (both increased in morbid obese patients). These adjusted correlation patterns offer a clear descriptive summary of metabolite co-variation independent of the modeled covariates.

### Classification performance via LDA

#### Univariate analysis

After selecting a concomitant metabolite (metabolite*_c_*) for each biochemical group, predicted concentrations of the remaining metabolites were estimated using linear models. For each subject and metabolite, excluding the concomitant itself, log_10_-transformed values were modeled as a function of age, sex, the interaction between age and sex, and the log_10_-transformed concentration of the concomitant metabolite. Residuals, defined as the difference between the observed metabolite levels and their predicted values, were then calculated. To avoid bias, the condition variable (obese/control) was not included in these models. This exclusion ensured that the residuals captured variation independent of condition status. By utilizing model-derived residuals instead of raw concentrations, this approach avoids reliance on fixed diagnostic thresholds and yields an individualized deviation from a subject-specific expected baseline. As such, it may be more informative and potentially more effective than classical concentration-based cut-offs, particularly when confounding factors and inter-individual lipidomic variability are substantial. In total, 127 residuals were obtained and merged across biochemical groups.

Each residual was first tested individually as a predictor for the condition in univariate linear discriminant analysis (LDA) models. These results demonstrated notable discriminative performance (Table 2), with the best performance achieved by LPC 17:0, yielding an accuracy of 0.78 and specificity of 0.75. The high accuracy of these residuals validates the selection of PC O-40:1 as a concomitant normalizer; although PC O- species often decrease in obesity, its role here as a baseline reference effectively isolated the pathological signal from the individual lipidomic background without introducing obesity-related bias. Performance metrics were quantified by cross-validation (see below), providing a rigorous internal estimate of generalization in this dataset. PC 38:0 also showed relevant classification capacity, with a specificity of 0.70. To achieve higher accuracies, combination of two or more metabolites may be required.

**Table 2:** Univariate discriminant analysis.

Glycerophospholipids	Sphingomyelins	AUC	Accuracy	Sensitivity	Specificity
**LPC 17:0**		0.77	0.78	0.75	0.875
**PC 38:0**		0.71	0.75	0.70	0.875
*PC 36:3*		0.71	0.72	0.70	0.750
*PC 32:1*		0.64	0.72	0.70	0.750
	*SM 18:1*	0.63	0.72	0.70	0.750
**PC 36:0**		0.64	0.68	0.70	0.625
**LPC 18:1**		0.72	0.64	0.65	0.625
**LPC 18:0**		0.65	0.64	0.65	0.625
**PC 40:4**		0.60	0.64	0.60	0.750
**LPC 18:2**		0.58	0.64	0.65	0.625

No amino acid or acylcarnitine molecule led to an accuracy greater than 0.6. The table includes the metabolic families of each metabolite and their trend in samples from obese individuals (bold: decrease, italic: increase).

#### Multivariate analysis: two to four metabolite combinations

To explore potential synergistic effects and enhance predictive performance, residuals were used in multivariate LDA models. All possible combinations of two, three, and four metabolites were tested, resulting in a total of approximately 10.7 million models (*n* = 10,676,001). This exhaustive search systematically assessed low-dimensional metabolite panels while capping model complexity at four predictors to ensure an appropriate bias–variance trade-off for the available sample size. This range was chosen to balance exploratory depth with clinical applicability, based on prior evidence suggesting that larger metabolite panels may not necessarily improve model utility. Model performance was assessed using Leave-One-Out Cross-Validation (LOOCV). LOOCV was chosen to maximize training data usage given the limited cohort size and to obtain approximately unbiased estimates of predictive performance. Table 3 summarizes the multivariate analysis of metabolite combinations (two, three, or four) that provided the highest specificities. Detailed results for each case are presented in Supplementary Tables S1–S3.

**Table 3:** Multivariate comparison.

Number of metabolites	Acyl-carnitines	Glycerophospho-lipids	Sphingomyelins	AUC^a^	Accuracy	Sensitivity	Specificity
2	-	**LPC 17:0** *PC 32:3*	-	0.84	0.82	0.75	1.00
3	-	**LPC 17:0** *PC 32:3*	*SM 16:1; O*	0.87	0.86	0.80	1.00
4	-	**LPC 17:0** *PC 32:3* *PC 36:2*	*SM 16:1; O*	0.87	0.86	0.80	1.00
4	*C2*	**LPC 17:0** *PC 32:3*	*SM 16:1; O*	0.86	0.86	0.80	1.00

a AUC: area under the ROC curve.

The table includes the metabolic families of each metabolite and their trend in morbid obesity (bold: decrease, italic: increase).

Supplementary Table S1 reports combinations of two metabolites with classification accuracy ≥0.82, together with their metabolic families and direction of change in obesity (increase in red, decrease in blue). The pair LPC 17:0 and PC 32:3 achieved the highest specificity, although sensitivity was limited to 0.75, resulting in an overall accuracy of 0.82. Comparable accuracy, but with a more balanced sensitivity/specificity ratio (0.91), was obtained with combinations of glutamic acid and LPC 17:0, as well as of PC 34:4 and PC 32:0.

Three-metabolite combinations moderately improved performance, with a maximum accuracy of 0.86 (Supplementary Table S2). Notably, LPC 17:0 was consistently included in all top-performing sets. Similarly, four-metabolite models yielded results comparable to those with three, with minimal gains in accuracy (Supplementary Table S3). Again, LPC 17:0 appeared in every best-performing combination. Regarding this candidate biomarker, LPC 17:0 was detected in 100% of samples and showed concentrations consistently above the limit of detection (LOD = 0.111 μM).

### Correlation with BMI and obesity stratification

The consistent presence of LPC 17:0 in all the best performing models justified a further investigation of residuals (using Equation 2, see Methods). Remarkably, a significant negative correlation with BMI across all subjects was shown. Pearson correlation yielded *r* = −0.59 (*p* = 0.001), and Spearman correlation yielded *ρ* = −0.63 (*p* = 0.0004), indicating that higher the BMI of a given patient is associated with lower residuals values of this metabolite.

When obese subjects were further stratified, Kruskal–Wallis tests revealed significant differences in LPC 17:0 residual values among the three groups (χ^2^ = 9.68, df = 2, *p* = 0.008). The Kruskal–Wallis test was used as a rank-based, distribution-free approach appropriate for comparing three groups without relying on normality or equal-variance assumptions. Post-hoc pairwise indicated that LPC 17:0 residual values were significantly lower in morbid obesity 2 compared to controls (adjusted *p* = 0.0023), while differences between morbid obesity 1 and controls, and between the two obesity subgroups, were not statistically significant after false discovery rate (FDR) correction of the *p*-values obtained from the pairwise post-hoc tests.

**Figure 3:**
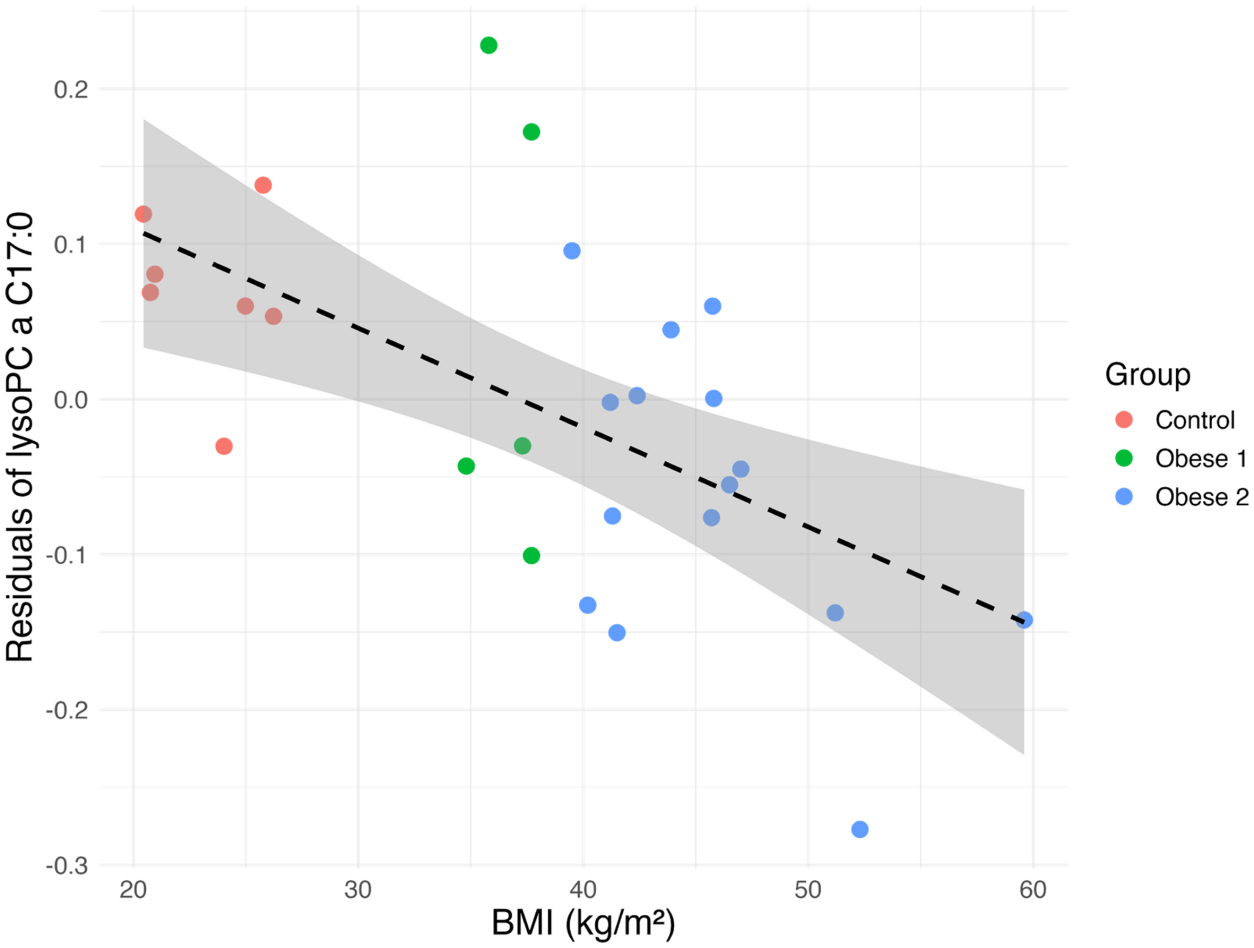
Correlation between BMI and predicted LPC 17:0 levels. Scatterplot of BMI (kg/m^2^) versus the residuals of LPC 17:0 calculated using Equation 2 across controls, morbid obesity type 1 (BMI ≥ 34.8 with comorbidities or missing comorbidity data), and morbid obesity type 2 (BMI ≥ 39.5). Points are colored according to group. The dashed black line indicates the overall linear regression trend, showing an inverse association between the two parameters.

These results suggest that LPC 17:0 residual values decrease progressively with increasing BMI, particularly in subjects at the highest end of the obesity spectrum (Figure 3), highlighting its potential as a marker of metabolic alterations associated with morbid obesity.

## Discussion

The clinical diagnosis of morbid obesity, while well-established through Body Mass Index (BMI) and co-morbidities criteria, would benefit from robust plasma biomarkers to enhance disease management, patient stratification and risk prediction. Plasma biomarkers are attractive due to their accessibility in routine clinical practice, but their reliability has often been limited by high interindividual variability in metabolite levels, as reported in previous metabolomics studies (21–24); they arise from genetics, diet, sex, age, and other factors. To address this limitation, a normalization strategy that considers these factors and is based on “concomitant” metabolites within biochemical families, previously developed in our laboratory (18), was applied in this study. By selecting a stable concomitant reference metabolite and extracting residuals adjusted for age, sex, and the concomitant metabolite, the background variability was reduced, and metabolites with robust discriminative power were identified. By adopting a residual-based approach rather than traditional static cut-offs, metabolic alterations are captured as significant deviations from a subject’s own predicted physiological baseline. This personalized strategy enables identification of a specific metabolic deficit (reflected by negative residuals) even when systemic concentrations are low, helping to distinguish pathological changes from inherent biological variability.

Correlation patterns within biochemical families provided initial insights (Figures 1; Supplementary Figures S1–S3), yet linear discriminant analysis (LDA) offered a more effective framework for biomarker selection. In the present cohort, LPC 17:0 demonstrated strong discriminative power on its own, with additional metabolites providing some improvements in classification. (Tables 2, 3). For comparison, applying the same framework to a cohort of Alzheimer’s disease patients required combinations of 3–4 metabolites to reach acceptable classification accuracy (19). This highlights the particularly strong discriminative power of LPC 17:0 in morbid obesity.

The results of this study align with recent lipidomics studies reporting reductions of multiple LPCs in obesity and related phenotypes, including normal-weight obesity and normal-weight central obesity, both associated with higher cardiometabolic risk (25). The consistent decrease of LPCs across cohorts from Asia, the Middle East, and Europe (26, 27) reinforces their role as cross-population signatures of adiposity-related dysfunction. In the current cohort, all measured LPCs were reduced in morbidly obese patients, further validating this pattern (Supplementary Figure S2).

PC O-40:1, selected as the concomitant metabolite for normalization, is a plasmalogen with antioxidant properties, particularly in heart and liver tissues (28). Beyond its role as a normalization reference, the coordinated reduction of LPC 17:0 within this lipid family suggests a dysregulation in lipid remodeling pathways, potentially linked to trace element exposure, excess body weight, and insulin resistance (29). Our findings specifically highlight LPC 17:0 as a cornerstone of this metabolic impairment. This metabolite has been previously identified as a robust biomarker strongly and negatively associated with morbid obesity (30). The clinical relevance of this odd-chain lipid is underscored by its dual role as both a metabolic shield and a systemic proxy for lifestyle factors. Primarily derived from dietary intake and gut microbiota fermentation, a reduction in LPC 17:0 may reflect gut dysbiosis and poor dietary quality (31), both of which are established drivers of obesity. Mechanistically, this depletion contributes to the development of type 2 diabetes (T2D) by removing the protective activation of the GPR120 receptor, which normally promotes GLP-1 secretion to revert hyperglycemia and insulin resistance (32). These functional insights provide a biological basis for the emerging evidence linking phosphatidylcholines to T2D. For instance, Mendelian randomization analyses have implicated specific phosphatidylcholines in the causal development of the disease (33), and interventional studies in obese mice suggest that supplementation with LPC 17:0 can improve metabolic profiles (32). Importantly, the findings of this analysis resonate with the longitudinal metabolomics study of diabetes progression (34), in which LPC 17:0 was associated with HbA1c level increase and served as a mediator of type 2 diabetes development over 18–48 months.

While our findings suggest promising molecular patterns, confirmation of these stratification approaches will clearly require validation in larger and independent cohorts. Limitations for this study include small sample size (20 obese patients and 10 controls, reduced to 28 after quality control), which limits statistical power and generalizability, although the strong signal of LPC 17:0 combined with PC O-40:1 suggests that larger cohorts could reinforce its biomarker potential. The cross-sectional design prevents longitudinal assessment of disease progression, highlighting the need for prospective validation. Plasma metabolomics is influenced by multiple factors such as diet, inflammation, and medication, but only sex and age were included as covariates. While this represents a limitation, it also reflects a pragmatic choice to ensure feasibility in clinical practice, where such basic information is consistently available, unlike more variable factors that are difficult to standardize. Finally, the current single-center cohort limits applicability across populations, underscoring the importance of multi-center replication.

Taken together, these data indicate that reductions in LPCs, particularly LPC 17:0 constitute a reproducible metabolic signature of morbid obesity. PC O-40:1 enhances comparability across studies as a reliable normalization partner. Longitudinal monitoring of these metabolites could enable monitoring of disease severity and identification of patients at highest cardiometabolic risk. This research contributes to the growing body of evidence supporting the clinical utility of metabolomics in the management of metabolic diseases and represents an important step toward implementing precision medicine approaches in obesity care. The identification of robust, clinically actionable metabolic biomarkers could transform the diagnostic, monitoring, and treatment.

## Materials and methods

### Samples

Plasma samples and associated clinical information from obese patients (*n* = 20) and healthy controls (*n* = 10) included in this study were provided by the Biobank I3PT,^1^ which belongs to the biobank network of the Spanish National Health Institute Carlos III; and is located within the Parc Taulí Hospital premises (Sabadell, Spain). Samples were obtained and processed following standard operating procedures with the appropriate approval of the Ethics and Scientific Committees.

### Analytical procedure and data collection

Targeted quantification employed the AbsoluteIDQ^™^ p180 Kit (Biocrates Life Sciences, Innsbruck, Austria), enabling measurement of 188 metabolites across biogenic amines, amino acids, hexoses, acylcarnitines, and phospho−/sphingolipids. The complete analyte panel is available on the manufacturer’s product page.^2^

Analyses were performed on an AB Sciex 6,500 QTRAP tandem mass spectrometer (AB Sciex LLC, Framingham, MA, United States) coupled to an Agilent 1,290 Infinity UHPLC (Agilent, Santa Clara, CA, United States). For HPLC–MS/MS, we used an Agilent ZORBAX Eclipse XDB C18 column (3.0 × 100 mm, 3.5 μm) at 50 °C. Mobile phases were water with 0.2% formic acid (A) and acetonitrile with 0.2% formic acid (B), with a constant flow of 0.5 mL·min^−1^. The gradient was: 0–0.5 min, 100% A; 0.5–5.5 min, linear to 95% B; 5.5–6.5 min, hold at 95% B; 6.5–7.0 min, return to 100% A; 7.0–9.5 min, re-equilibrate at 100% A. MS operated with positive-mode ESI (probe x = 8, y = 0), and data were acquired in MRM using kit-supplied transitions. For the metabolites detected using this approach, retention time and mass transitions, are provided in Supplementary File.

For FIA–MS/MS, the carrier was methanol containing the kit’s FIA Mobile Phase Additive. The flow program was: 1.6 min at 0.03 mL·min^−1^; ramp over 0.8 min to 0.20 mL·min^−1^; hold 0.4 min; decrease to 0.03 mL·min^−1^ over 0.2 min. ESI was run in positive mode (probe x = 5, y = 5) with MRM acquisition as per the kit.

Sample preparation followed the manufacturer’s guidance. Briefly, each well received 10 μL of internal standard (kit-provided) plus either 10 μL PBS (blank), 10 μL calibration standards, 10 μL quality controls (kit), or 10 μL of plasma samples. Plates were dried at ambient temperature under nitrogen, then derivatized with 5% (v/v) phenylisothiocyanate (PITC) in ethanol:water:pyridine (1,1,1). After 20 min at room temperature, wells were dried again under nitrogen and reconstituted in 300 μL extraction solvent (5 mM ammonium acetate in methanol). Prior to acquisition, aliquots were diluted 1:1 with Milli-Q water for HPLC–MS/MS or 1:10 with FIA mobile phase for FIA–MS/MS.

Data processing used Analyst (AB Sciex LLC, Framingham, MA, United States) and MetIDQ^™^ (Biocrates Life Sciences, Innsbruck, Austria).

To ensure high analytical robustness and reproducibility, the AbsoluteIDQ^™^ p180 platform incorporates class-specific internal standards. This technical setup, combined with the sensitivity of the tandem mass spectrometer, allows for an effective distinction between biological signals and analytical noise, ensuring reliable quantification across the measured lipidomic profile.

### Statistical analysis

Statistical analyses were performed in *R* version 4.4.3 using *RStudio* (version 2024.09.0 + 375) and several packages, including *dplyr* (v1.1.4) and *tidyr* (v1.3.1) were employed for the analysis. All analyses were executed via fully scripted, version-controlled workflows, with exact package versions recorded to ensure complete computational reproducibility.

The initial dataset for the following analysis included quantitative measurements for 188 metabolites across 30 plasma samples, together with demographic and clinical variables such as age, sex, body mass index (BMI), and comorbidities. The study population consisted of 10 control subjects and 20 obese subjects. The mean age was 40.0 ± 4.7 years in the control group (5 females, 5 males) and 53.3 ± 6.1 years in the obese group (11 females, 9 males), with ages ranging from 31 to 45 years and from 42 to 63 years, respectively. Obese individuals were defined by body mass index (BMI) ≥ 34.8 kg/m^2^, whereas controls had BMI < 26.23 kg/m^2^ and no known metabolic disorders. Among obese subjects, some metabolic comorbidities were present, including type 2 diabetes, hypertension, dyslipidemia and sleep apnea syndrome.

Preprocessing was performed to ensure data reliability. First, metabolites with more than 20% of values “below the limit of detection (LOD)” across samples were excluded, reducing the dataset from 188 to 139 metabolites. Subsequently, a quality control check was conducted to identify and potentially exclude individual samples with excessively low-abundance readings. At the sample level, a quality control filter was applied to remove any sample with >30% missing metabolite concentrations, but none met this criterion. Remaining missing values, corresponding to concentrations below the LOD, were imputed as LOD/2. This standard targeted-metabolomics imputation yields conservative low-end estimates while maintaining stable variance properties suitable for small-sample analyses. Outlier detection was then performed through principal component analysis (PCA) on the scaled metabolite matrix using the *prcomp* function in *R*. The first two principal components (PC1 and PC2) were used to calculate Mahalanobis distances for each sample, and a chi-squared cutoff at the 97.5th percentile (df = 2) was applied to define outliers. This step led to the exclusion of two samples, leaving a final set of 28 plasma samples (20 obese patients and 8 controls). To visualize the multivariate structure of the final dataset and assess the metabolic separation between groups, a secondary exploratory PCA was performed on the pre-processed and Unit Variance (UV) scaled data. Confidence ellipses (95%) were calculated to represent the distribution of each clinical group in the reduced feature space. Importantly, these ellipses were used exclusively for visualization; outlier identification was based solely on the Mahalanobis distance thresholds defined during the initial data-cleaning phase (computed in the original feature space).

Subsequently, to preserve biological interpretability while reducing data dimensionality, metabolites were assigned to biochemical groups according to the classification provided in the AbsoluteIDQ^®^ p180 targeted metabolomics kit (Biocrates Life Sciences AG, Innsbruck, Austria). This resulted in five major categories: acylcarnitines, amino acids, biogenic amines, glycerophospholipids, and sphingomyelins. The parameter H1, which correspond to the sum of the level of all hexoses was excluded. The final dataset used for downstream analyses consisted of 138 metabolites across the five biochemical groups.

### Determination of the concomitant metabolite for each biochemical family

Within each biochemical group, a series of linear models was constructed to select the best concomitant metabolite (metabolite_c), which was included as a covariate in the models. For each metabolite pair belonging to the same biochemical group (indicated as predicted metabolite and metabolite_c), a linear model was created. A linear model of the following form (1) was fitted for each metabolite *j* across samples *i*:


$$\begin{array}{l} \log_{10}{\left[\text{metabolit}e_{\mathrm{ij}}\right]}_{\text{predicted}}=\beta_{0j}+\beta_{1j}\cdot \mathrm{Ag}e_{i}+\beta_{2j}\cdot \mathrm{Se}x_{i}+\\ \beta_{3j}\cdot (\mathrm{Ag}e_{i}\times \mathrm{Se}x_{i})+\\ \beta_{4j}\cdot \text{Conditio}n_{i}+\\ \beta_{5j}\cdot \log_{10}[\text{metabolite}\_c_{i}]+\varepsilon_{\mathrm{ij}}\; \end{array}$$ (1)

Details of the procedure that was developed in our laboratory can be found elsewhere (18). One of the clues of the approach is to include demographic covariates, in the present case: age, sex and their interaction (Age×Sex). Normalization is performed by calculating the *predicted* value from the actual concomitant concentration and applying Equation 1.

Concomitant metabolites were selected according to multiple statistical criteria: (i) the average coefficient of determination (*R^2^*) across models in which the metabolite was included, (ii) the overall significance of the model (F-test), and (iii) the statistical significance of the metabolite*_c_* coefficient (*p*-value). These criteria were applied uniformly across biochemical families to prioritize metabolites with consistent explanatory contribution and stable model behavior while keeping model complexity aligned with the available sample size. The metabolite with the strongest overall performance within each group was selected as the concomitant metabolite (metabolite*_c_*).

### Correlation heatmaps

Once a concomitant metabolite was selected for each biochemical group, the same linear model (1) was applied again, this time focusing on residual extraction.

Residuals (*e_ij_*) were calculated using this equation:


$$e_{\mathrm{ij}}=\log_{10}{\left[\text{metabolit}e_{\mathrm{ij}}\right]}_{\text{observed}}-\log_{10}{\left[\text{metabolit}e_{\mathrm{ij}}\right]}_{\text{predicted}}$$ (2)

Where the predicted values, calculated using Equation 1, represent the variation explained by age, sex, condition, and the concomitant metabolite, while residuals captured the unexplained variability. These residuals were used to investigate the relationships between metabolites independently of the known sources of variation. Further details of this pipeline are indicated in Stefanini et al. (19).

### Linear discriminant analysis (LDA)

After identification of the concomitant metabolite for each biochemical group, the same modeling strategy (1) was applied again to all metabolites in the group (excluding the concomitant metabolite, metabolite*_c_*), this time removing the condition variable from the formula to avoid bias.

Residuals derived from this model represented the component of metabolite variability not explained by age, sex, or the concomitant metabolite. The residuals were used as predictor variables in linear discriminant analysis (LDA) models to classify subjects as obese or control. This residual-based approach emphasizes departures from each subject’s predicted baseline rather than fixed cut-offs, helping accommodate inter-individual metabolic variability. Accordingly, negative residuals indicate lower-than-expected levels after adjusting for age, sex, and the concomitant metabolite.

Given the imbalance in the dataset (20 obese vs. 8 controls), the classification threshold for posterior probability was adjusted to the proportion between obese and control participants (0.714). LDA-related computations and plot generation were performed using in *R*, and the model performance was evaluated using LOOCV. LOOCV was used to exploit the full dataset for model fitting in each iteration and to provide a rigorous internal estimate of out-of-sample predictive performance in this cohort.

### Correlation with BMI and obesity stratification

To further investigate the relevance of the most recurrent metabolite, its association with body mass index (BMI) across all subjects was evaluated. Pearson and Spearman correlation tests were applied to assess linear and monotonic relationships, respectively.

Additionally, the obese cohort was stratified into two subgroups based on BMI and comorbidity status to explore potential gradients of metabolite alterations. Subjects were classified as Type 1 morbid obesity if BMI ≥ 34.8 kg/m^2^ with either presence of comorbidities or missing comorbidity data, and as Type 2 morbid obesity if BMI ≥ 39.5 kg/m^2^.

Non-parametric Kruskal–Wallis tests were applied to compare metabolite levels across the three groups (controls, Type 1 morbid obesity, and Type 2 morbid obesity). Post-hoc pairwise comparisons were performed using Wilcoxon rank-sum tests with false discovery rate (FDR) correction.

Data handling, statistical tests, and visualization were performed using dplyr (v 1.1.4), ggplot2 (v3.5.2), ggpubr (v 0.6.1), and stats (v 4.5.1) packages.

## Data Availability

The original contributions presented in the study are included in the article/Supplementary material, further inquiries can be directed to the corresponding author.

## Footnotes

1
https://www.isciiibiobanksbiomodels.es/centros/biobanc-i3pt/

2
https://biocrates.com/products/research-products/absoluteidq-p180-kit
