# Supplementary material for: Metabolomics reveals LysoPC a C17:0 (LPC 17:0) as candidate biomarker for personalized medicine in morbid obesity
Source: Front Med (Lausanne). 2026 Mar 30;13:1779275. doi: 10.3389/fmed.2026.1779275 (PMC13071009; doi:10.3389/fmed.2026.1779275)
Supplement: Supplementary file 1 [file Supplementary_file_1.pdf]

Supplementary Material 1/2 (Figures and tables)

Figures S1, S2 and S3 and Tables S1, S2 and S3.

Metabolomics Reveals LysoPC a C17:0 (LPC 17:0) as Candidate Biomarker for Personalized Medicine in Morbid Obesity

Eleonora Stefanini [1], Silvia Marín[1,2,3], Joan Serrano-Marín [1*], Juan Sánchez-Navés [4], Hanan A. Alkozi [5], Mercè Pallàs [6,7,8], Marta Cascante[1,2,3], Christian Griñán-Ferré [6,7,8] and Rafael Franco [1,8,9,*]

1. Department of Biochemistry and Molecular Biomedicine, Universitat de Barcelona, 08028 Barcelona, Spain.

2. Institute of Biomedicine of University of Barcelona (IBUB), University of Barcelona (UB), Barcelona, Spain.

3. CIBEREHD, Network Center for Hepatic and Digestive Diseases, Spanish National Health Institute Carlos III (ISCIII), Madrid, Spain

4. Department of Ophthalmology, Oftalmedic, I.P.O. Institute of Ophthalmology, 07011 Palma de Mallorca, Spain.

5. Department of Optometry, College of Applied Medical Sciences, Qassim University, Buraydah 51452, Saudi Arabia.

6. Departament de Farmacologia i Química Terapeùtica, Universitat de Barcelona, 08028 Barcelona, Spain;
pallas@ub.edu (M.P.); christian.grinan@ub.edu (C.G.-F.)

7. Institut de Neurociències, Universitat de Barcelona, 08035 Barcelona, Spain

8. CiberNed, Network Center for Neurodegenerative Diseases, Spanish National Health Institute Carlos III (ISCIII), 28029 Madrid, Spain

9. Institut of Computational Chemistry o(IQTC) of the University of Barcelona School of Chemistry, Universitat de Barcelona, 08028 Barcelona, Spain

* Corresponding authors: rfranco123@gmail.com or rfranco@ub.edu ; joan.serrano.marin@gmail.com

FIGURES S1, S2 and S3

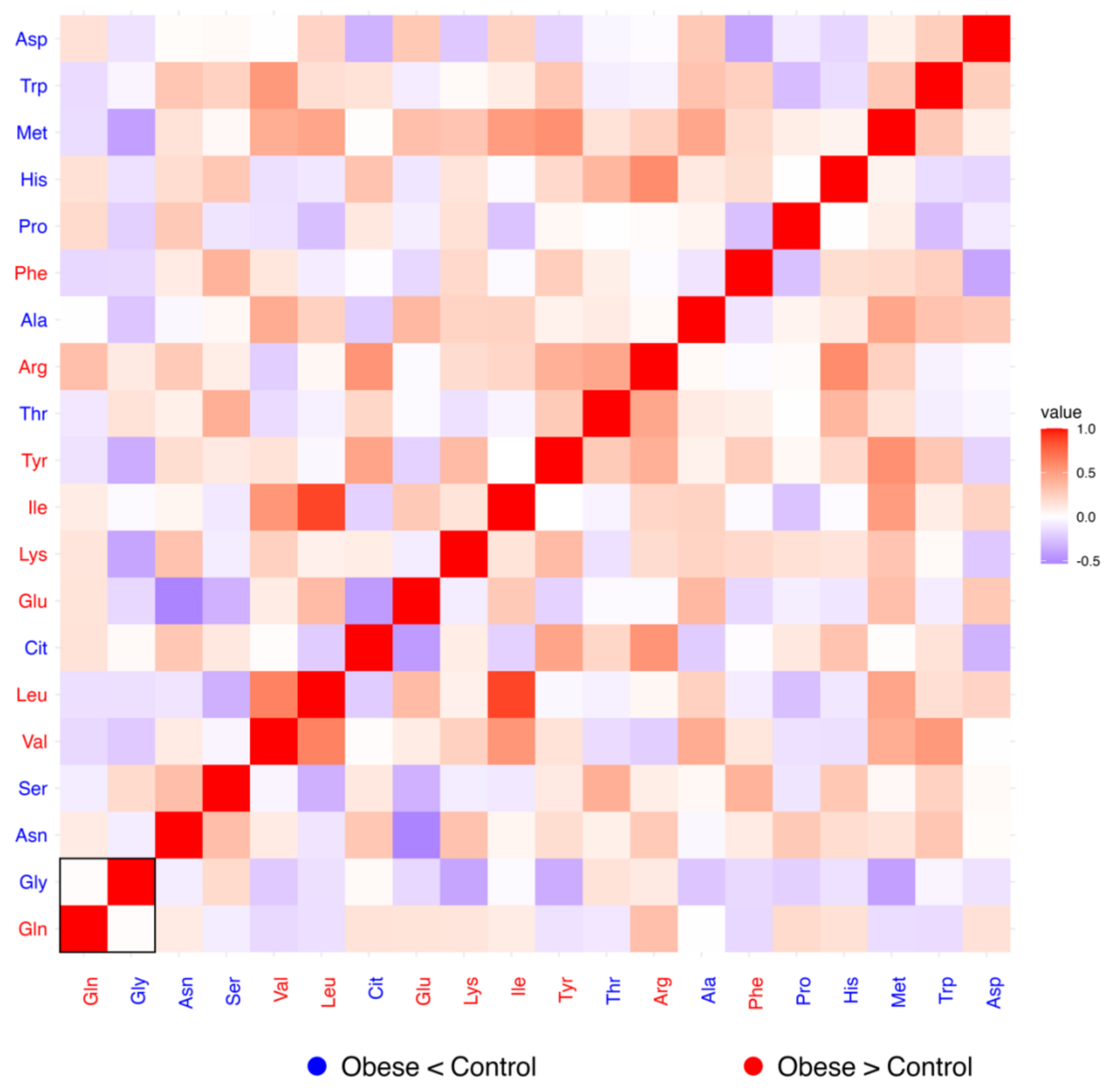

Figure S1. Heatmap of residual correlations within the of amino acids group. *Metabolite_c* for this family was ornithine. Blue labels indicate tendency to decrease in obesity and red labels indicate a tendency to increase in obesity. Red cells indicate positive correlation (1 means perfect correlation), blue cells indicate negative correlation, and white cells indicate no correlation. $p < 0.05$ for residuals of metabolites within the solid line frame.

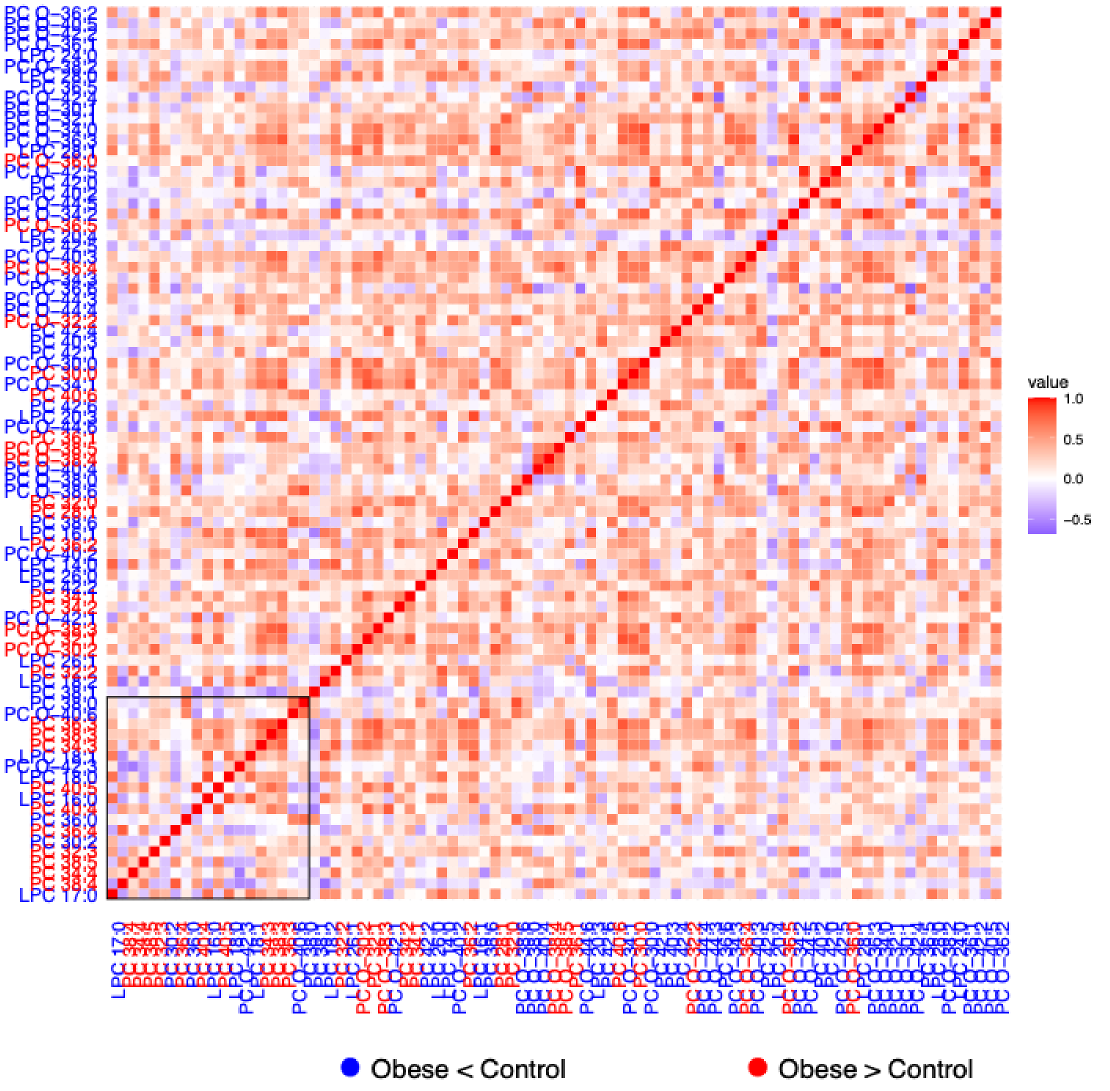

Figure S2. Heatmap of residual correlations within the glycerophospholipid group. *Metabolite_c* for this family was PC O-40:1. Blue labels indicate tendency to decrease in obesity and red labels indicate a tendency to increase in obesity. Red cells indicate positive correlation (1 means perfect correlation), blue cells indicate negative correlation, and white cells indicate no correlation. $p < 0.05$ for residuals of metabolites within the solid line frame.

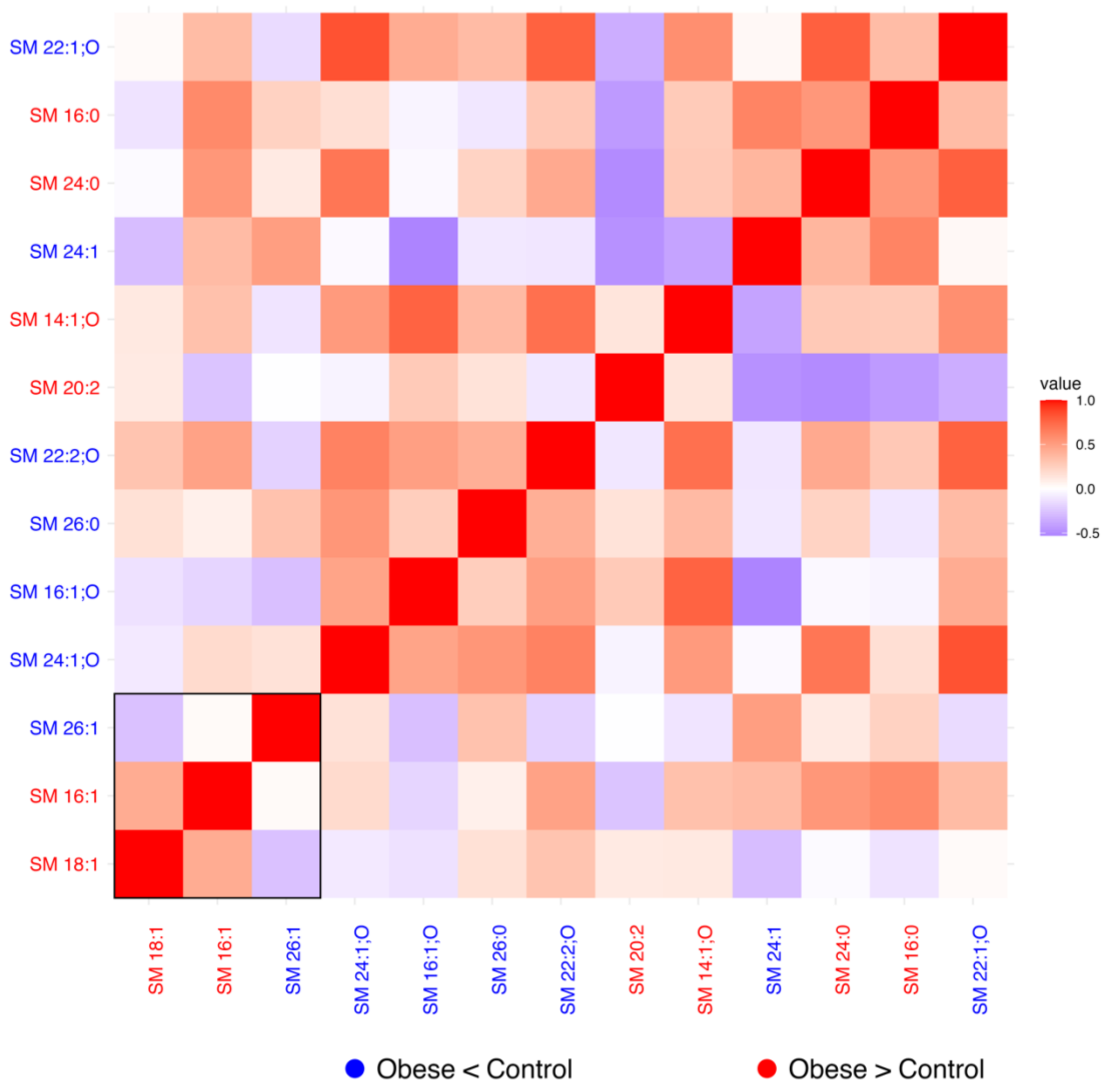

Figure S3. Heatmap of residual correlations within the sphingomyelins group. *Metabolite_c* for this family was SM 18:0. Blue labels indicate tendency to decrease in obesity and red labels indicate a tendency to increase in obesity. Red cells indicate positive correlation (1 means perfect correlation), blue cells indicate negative correlation, and white cells indicate no correlation. $p < 0.05$ for residuals of metabolites within the solid line frame.

Tables S1, S2 and S3

Table S1. Best results of multivariate discriminant analysis using the residuals of two metabolites as predictors.

Acylcarnitines	Amino Acids	Glycerophospholipids	Sphingomyelins	AUC	Accuracy	Sensitivity	Specificity
-	-	LPC 17:0<br>PC 32:3	-	0.84	0.82	0.75	1.00
-	Glu	LPC 17:0	-	0.74	0.82	0.80	0.875
-	-	PC 34:4<br>PC 32:0	-	0.72	0.82	0.80	0.875

Table S2. Best results of multivariate discriminant analysis using the residuals of three metabolites as
predictors.

Acylcarnitines	Amino Acids	Glycerophospholipids	Sphingomyelins	AUC	Accuracy	Sensitivity	Specificity
-	-	LPC 17:0<br>PC 32:3	SM 16:1;O	0.87	0.86	0.80	1.00
C2	-	LPC 17:0<br>PC 32:3	-	0.86	0.86	0.85	0.875
-	-	LPC 17:0<br>PC 32:3<br>PC 34:2	-	0.86	0.86	0.80	1.00

Table S3. Best results of multivariate discriminant analysis using the residuals of four metabolites as predictors.

Acylcarnitines	Amino Acids	Glycerophospholipids	Sphingomyelins	AUC	Accuracy	Sensitivity	Specificity
-	-	LPC 17:0<br>PC 32:3<br>PC 36:2	SM 16:1;O	0.87	0.86	0.80	1.00
C2	-	LPC 17:0<br>PC 32:3	SM 16:1;O	0.86	0.86	0.80	1.00
C2<br>C3	-	LPC 17:0<br>PC 32:3	-	0.86	0.86	0.85	0.875
